# Novel Case of Bilateral Adrenal Tumors Confirms Pathogenicity of Previously Described c.463+4C>G Variant in the von-Hippel Lindau Gene

**DOI:** 10.15586/jkc.v12i1.381

**Published:** 2025-03-01

**Authors:** Samuel Morriss, Victoria Beshay, Huei San Leong, Ingrid Winship

**Affiliations:** 1 Familial Cancer Centre, The Royal Melbourne Hospital, Parkville, Victoria, Australia;; 2 Peter MacCallum Cancer Centre, Melbourne, Victoria, Australia;; 3 Faculty of Medicine, Dentistry and Health Sciences, University of Melbourne, Carlton, Victoria, Australia

**Keywords:** adrenal tumors, pathogenic variant, pheochromocytoma, reduced penetrance, von Hippel-Lindau (VHL)

## Abstract

We report a case of a pathogenic variant c.463+4C>G in the von Hippel-Lindau (VHL) gene identified in a patient presenting with bilateral adrenal tumors, including a histologically confirmed pheochromocytoma with no significant family history of VHL-associated tumors. This same variant was first reported as having pathogenic significance in an unrelated proband with a hemangioblastoma and a family history of pheochromocytoma. In our patient, next-generation sequencing and subsequent RNA (ribonucleic acid) analysis confirmed this mutation to be a pathogenic (class 4) variant in intron 2. The lack of family history of VHL-associated tumors correlated with the proband further suggests that this mutation may have reduced penetrance. This case confirms the pathogenicity of the same previously described variant in the VHL gene and underscores the utility of genetic testing in patients with atypical presentations of adrenal tumors, even in the absence of a relevant family history.

## Introduction

Von Hippel-Lindau (VHL) disease is an autosomal dominant condition characterized by multiorgan cysts and tumors. These include hemangioblastomas of the central nervous system and retina, renal cell carcinoma, endolymphatic sac tumors, epididymal and broad ligament cystadenomas, pancreatic cysts, and neuroendocrine tumors such as pheochromocytomas and paragangliomas (1). The tumor suppressor gene VHL is the only gene implicated thus far in the pathogenesis of VHL, whereby a single inherited mutant VHL allele along with a second hit in the wild-type copy may result in tumor development (2). Furthermore, sporadic somatic mutations of the VHL gene has been seen in *de novo* VHL-associated tumors (3).

This report outlines a pathogenic variant in the VHL gene associated with bilateral adrenal tumors, with one being a histologically confirmed pheochromocytoma. This variant is identical to a previously reported novel variant sequenced in an unrelated family with a history of hemangioblastoma and pheochromocytoma (4).

## Case presentation

A 29-year-old female was referred by her urologist for the genetic opinion of bilateral adrenal tumors. She had initially experienced symptoms of facial flushing and occasional palpitations without hypertension or tachycardia. There was no other past medical history. She was referred to an endocrinologist for further assessment. Plasma normetanephrine levels exceeded 6000 pmol/L (reference range < 900 pmol/L). A right suprarenal mass was also seen on ultrasound. This was further characterized with computed tomography (CT) of the kidneys and adrenal glands, revealing a cystic-appearing right suprarenal lesion and a smaller contrast-enhancing lesion in the left adrenal gland. Both lesions were suspicious for pheochromocytomas. The patient went on to receive a Gadolinium (Ga)-68 DOTATATE positron emission tomography (PET)-CT which demonstrated an avid cystic mass arising from the right renal gland; the left adrenal lesion did not demonstrate abnormal DOTATATE activity.

The patient subsequently underwent a right robotic adrenalectomy to excise a cystically enlarged adrenal gland measuring 100 × 75 × 50 mm. Histology revealed a moderately differentiated pheochromocytoma with a Grading of Adrenal Pheochromocytoma and Paraganglioma (GAPP) score of 3, indicating a moderately differentiated adrenal medullary tumor. Tumor cells were found to be intermingling with adrenal cortical cells, with Ki-67 (Antigen Kiel 67) labelling reported as 2% in some areas. There was no increased cellularity, obvious mitoses, or comedo-type necrosis identified and no presence of capsular or vascular invasion. SDHB (succinate dehydrogenase [ubiquinone] iron-sulfur subunit, mitochondrial) expression was retained, with immunohistochemistry showing a granular cytoplasmic staining.

The patient remained asymptomatic from a sympathetic nervous system hyperactivity perspective post removal of the right-sided pheochromocytoma, but despite this, plasma normetanephrine levels remained elevated at 1311 pmol/L. A repeat Ga-68 DOTATATE PET-CT demonstrated the left adrenal lesion to be stable in size at 21 mm with low-grade avidity, making pheochromocytoma equivocal. The patient has since been referred to an endocrine surgeon for consideration of a partial left adrenalectomy.

There was no known family history of VHL disease, neuroendocrine, or other inherited conditions. The family history obtained revealed a history of breast cancer, papillary urothelial cancer of the bladder, and thyroid cancer. There was no consanguinity within the family.

There is no known association between VHL and breast cancer reported in the literature (5), and also no reported associations between VHL and urothelial cancer of the bladder apart from the established phenotype of clear cell renal cell carcinoma seen in VHL (1). Some studies suggest an association between VHL and papillary thyroid carcinoma (6–8).

### Molecular analysis

The patient consented to genetic testing for a VHL variant alongside the next-generation panel testing for mutations in a series of other genes associated with pheochromocytoma (i.e., EPAS1, FH, IDH1, KIF1B, KIT, MAX, MDH2, NF1, NF2, PDGFRA, RET, SDHA, SDHAF2, SDHB, SDHC, SDHD, and TMEM127). VHL gene sequencing revealed a likely pathogenic (class 4) variant c.463+4C>G in intron 2, and in silico analysis of this single nucleotide substitution predicted possible effects on splicing.

This exact variant was published in 2015 in a proband with a cerebral hemangioblastoma and a family history of pheochromocytoma and hemangioblastoma (4). There is no likelihood of any familial link between the two families as they have distinct ancestral backgrounds. RNA studies using semi-quantitative reverse transcription-polymerase chain reaction (RT-PCR) in this patient demonstrated that this variant resulted in skipping exon 2 and a reduction in the amount of transcript encoding of the full protein containing exons 1–3 (approximately 60%) compared to normal controls.

## Discussion

The VHL gene encodes the VLH protein (pVHL) that has roles in the regulation of hypoxia-inducible transcription factors (HIFs) via ubiquitination, apoptosis and senescence, extracellular matrix and cilia formation, cytokine signaling, and transcriptional regulation (2, 9). Pathogenic variants of the VHL gene result in either decreased expression of VHL or expression of a missense variant that is more prone to protein misfolding and subsequent degradation (10). Cells lacking sufficient functional pVHL shift the cell metabolic phenotype from oxidative phosphorylation to glycolysis as described by the Warburg effect (11). Furthermore, the apoptotic ability of cells is affected, which may explain the predisposition toward the development of VHL-associated tumors (2). Deficiency in pVHL has also been implicated as a mechanism of oncogene addiction due to the resultant activation of proto-oncogenes (12).

It has been previously documented that this variant in VHL c.463+4C>G may be associated with reduced penetrance, as evidenced by the heterogeneous phenotype seen in different family members with the mutation (4). VHL is not highly penetrant at an early age (under 65 years old). In the family where this variant was first identified, five mutation positive and seven untested family members at 50% risk of inheriting the variant were found to be negative for VHL-associated tumors (4). It was hypothesized that residual amounts of semi-functional protein may provide enough compensatory function to prevent development of the full VHL phenotype in affected individuals (4).

The VHL gene is well conserved in vertebrates, including humans, mice, rats, zebrafish, and chickens (1). Exon 2 of the VHL gene comprises almost the entirety of the nuclear export function region of the β-domain and is critical for known VHL function (13). Exon 2-encoded β--domain plays two independent roles in binding to HIFα and fibronectin (substrate recognition) and mediating -transcription-dependent nuclear/cytoplasmic trafficking of the VBC/Cul-2 complex (14).

There have been multiple reported instances of patients with VHL variants resulting in exon 2 skipping and with those individuals exhibiting variable phenotypes. One study reported a synonymous variant in exon 2, c.429C>T p. (Asp143=), which had a weaker impact on splicing compared to a different synonymous variant c.414A>G p. (Pro138=), and the carriers presented with autosomal recessive erythrocytosis (15, 16). The c.414A>G p. (Pro138=) carriers, however, had VHL disease with variable phenotypes, including three asymptomatic carriers in one family, with one being a woman aged 84 years old (16).

A recent study has shown several missense variants in exon 2 that resulted in upregulation of the exon 2 skipping splice variant, in which the carriers presented with variable phenotypes with two families diagnosed as Type 1 (low risk of developing pheochromocytoma) and the other two diagnosed as Type 2 (high risk of developing pheochromocytoma) (17); some of the carriers were reported to be unaffected. Collectively, this suggests that some VHL mutant alleles are not fully penetrant.

The case report first documenting this VHL c.463+4C>G variant highlights that this variant was initially characterized as a variant of unknown significance (4). This was subsequently reclassified to a likely pathogenic variant due to further analysis revealing reduction in VHL transcripts, predictions of effect on splicing, no reported SNPs (single nucleotide polymorphisms) at that particular nucleotide position, and other family members with the variant demonstrating similar phenotypes to the proband (4).

This case study of the same variant lends further evidence toward this variant being pathogenic. The variable expressivity and penetrance of this mutation is evidenced by comparing this patient’s phenotype of bilateral adrenal tumors diagnosed at 28 years of age with that of the original reported case study by which the proband initially presented with a cerebral hemangioblastoma at 34 years of age. The differences in family history between the two probands also highlight the likely variable penetrance of this mutation.

## Conclusion

This case report highlights a pathogenic variant in the VHL gene c.463+4C>G associated with bilateral adrenal tumors and a histologically proven pheochromocytoma. This finding contributes to the growing body of evidence supporting the pathogenicity of this variant in the context of VHL. The patient’s presentation and subsequent genetic analysis underscore the importance of genetic testing in individuals with atypical presentations of adrenal tumors, even in the absence of a family history of VHL-associated conditions.
